# Does knowledge on socio-cultural factors associated with maternal mortality affect maternal health decisions? A cross-sectional study of the Greater Accra region of Ghana

**DOI:** 10.1186/s12884-019-2197-7

**Published:** 2019-01-28

**Authors:** Lily Yarney

**Affiliations:** Department of Public Administration & Health Services Management, University of Ghana Business School, Accra, Ghana

**Keywords:** Socio-cultural factors, Maternal mortality, Maternal health decision, Ghana

## Abstract

**Background:**

The concern of all maternal health stakeholders is to improve maternal health and reduce maternal deaths to the barest minimum. This remains elusive in low and middle-income countries as the majority of factors that drive maternal deaths stem from the socio-cultural environment especially in rural settings. This study was aimed at finding out if knowledge on socio-cultural factors related to maternal mortality affects maternal health decisions in rural Ghana.

**Methods:**

Community-based cross-sectional in design, the study involved 233 participants from 3 rural districts in the Greater Accra Region. Mixed-method of data collection was employed after informed consent. Quantitative data were analyzed using simple statistics, Fisher’s Exact Test of independence and crude odds ratio were used to interpret the results, whilst the FGDs were recorded, transcribed and analyzed based on themes.

**Results:**

Statistically, significant relationship exists between all the socio-cultural factors studied (Traditional Birth Attendants (TBAs), religious beliefs and practices, herbal concoctions, and pregnancy and childbirth-related taboos) and maternal health decisions (*p* = 0.001 for all the variables) with very strong associations between maternal health decisions and knowledge on pregnancy and childbirth related taboos, TBA patronage, and religious beliefs and practices (OR = 21.06; 13; 7.28 respectively). However, misconceptions on factors associated with maternal mortality deeply rooted in rural communities partly explain why maternal morbidity and mortality are persistent in Ghana.

**Conclusion:**

Meaningful and successful interventions on maternal mortality can only be achieved if misconceptions on causes of maternal mortality especially in rural areas of the country are tackled through mass education of communities. This should be done consistently over a long period of time for sustained behavioral change.

**Electronic supplementary material:**

The online version of this article (10.1186/s12884-019-2197-7) contains supplementary material, which is available to authorized users.

## Background

In spite of all the effort by various countries and organizations to stem its tide, maternal mortality is still appreciably high in low and middle-income countries. In 2010 alone, 287,000 women lost their lives during and following pregnancy and childbirth [1]. Statistics indicate that 99% of the world’s maternal deaths occur in low and middle-income countries, out of this 85% come from sub-Saharan Africa and South Asia [2]. Even in a mixed population like the United States of America, the percentage of non-white women who die as a result of little or no antenatal care is more than three times what obtains in their white counterparts with the same exposure [3].

The lifetime risk of a woman dying out of childbirth in Ghana is 1 in 68 [1], and maternal mortality occurs all over the country irrespective of the number of health institutions and trained personnel available. Maternal mortality figures in 2013 indicate an urgent need to effectively tackle the problem. According to the UN agencies report released on 6th May, 2014, a total of 3100 maternal deaths were recorded from January to December, 2013 (with a maternal mortality ratio of 380 per 100, 000 live births). This may even be the tip of the iceberg, since a lot of maternal deaths occur as a result of induced illegal abortions. Additionally, in the Ghanaian context, domiciliary delivery outnumbers institutional delivery, the majority of these deaths therefore go unreported [4]. This probably explains the wide disparity between the institutional figure of 1012 and that of the UN agencies report in the same year.

Identifying the causes of maternal mortality is a first step in dealing with maternal health holistically. However, understanding the socio-cultural determinants of maternal deaths and their inter-relationships, may be paramount to developing effective preventive measures and remedies that target high-risk individuals and groups [4] as well as drawing up policies for effective intervention programs [5].

The causes of maternal mortality are well known, and have been categorized broadly as clinical or medical, and socio-cultural. The former includes hemorrhage, anemia, obstructed labor, abortion (voluntary and involuntary), sepsis, hypertensive disorders, cardiac disorders, ectopic pregnancies, renal failure, viral hepatitis, and malaria [6–9]. The interventions for these are based on medical explanatory model, thus, with timely diagnoses and well-resourced healthcare systems in place, the outcome of saving the mothers is obviously good. However, the factors that precipitate illness and death, or promote health are not solely biological, and can be cultural, social, psychological and economical. Thus, three decades after the Nairobi Conference in 1987, and other major conferences such as the 1990 World Summit for Children, the 1994 Cairo International Conference on Population and Development, and the 1995 Fourth World Conference on Women, all of which had the goal of halving the 1990 maternal mortality figures by the year 2000, maternal mortality ratios are still high in some parts of the world. Ghana signing up on the Nairobi declaration put up various interventions which included free maternal care, establishment of maternal and child health clinics, safe motherhood protocol development, training of traditional birth attendants among others. The Nairobi conference was an eye opener that paved the way for the development of the major maternal health related policies and interventions in the country. With these policies and interventions in place, Ghana’s maternal mortality ratio dropped from 760 per 100,000 live births in 1990 to 570 per 100,000 live births in 2000 [10], though Ghana was not able to halve her maternal deaths by the year 2000 [11]. With Ghana signing up on the Millennium Development Goals (MDGs) in 2000 and therefore committed to achieving the MDG 5 by reducing her maternal mortality by 75 % by 2015, the country made a recognizable gain by reducing the 1990 maternal mortality ratio figure to 319 deaths per 100,000 live births in 2015 representing an overall reduction of 44% in fifteen years. It is however, clear that safe motherhood interventions have not lived up to expectation since maternal mortality ratios in Ghana and other low and middle-income countries are still high. Some scholars attribute this to the failure on the part of policy makers to identify and tackle effectively the other category of determinants- socio-cultural [4].

Socio-cultural practices and factors that influence maternal deaths in Ghana have been identified to include low socio-economic status of women, early marriage and childbearing, poor dietary practices and taboos surrounding pregnancy, use of trained and untrained Traditional Birth Attendants (TBAs), induced abortion [4], and ‘the three delays’ (which usually occur during labor and delivery) that have their roots from the social, economic and cultural environment. These delays are: recognition of obstetric problems, decision to take pertinent steps to act on the problem, and arrival at appropriate health facility for intervention [12]. In addition, a fourth delay has been identified in Ghana: the health facility that may be finally accessed. The health facility may lack competent personnel, relevant equipment and logistics, and essential utility services such as reliable water supply and electricity [4]. Causes of maternal mortality in Ghana can best be understood with the application of medical anthropology which explains how people of different cultures and social status view and perceive illnesses and their cure [4]. In Ghana for example, local contextual issues influence the paradigm to disease causation and the health-seeking behavior of Ghanaians. Thus, to understand why many rural women patronize the services of TBAs, interpretivist approach to research may be the best way to go.

Women in Ghana like women in other countries such as Nigeria and Ethiopia prefer to be attended to by elderly women or TBAs during child birth [13]. This really poses risk to such women, as birth complications are always very difficult to deal with even by highly qualified health professionals. The question to ask is: do these women know the risk that these socio-cultural practices and critical decisions they take during their childbearing age pose to their lives?

In 2013, the Greater Accra Region of Ghana recorded a sizeable number of maternal deaths of 201 out of the 1012 institutional maternal deaths countrywide according to the report released by the Ghana Health Service (GHS), in March 2014. This is quite strange because this region has one of the highest geographical access to quality maternal health care. What then prevents inhabitants from using these facilities? Perhaps the answers are found in related literature. Social and cultural factors have been highlighted by various scholars as indispensable when dealing with maternal deaths holistically especially in low and middle-income countries [4, 14]. Socio-cultural factors have therefore been identified as having significant influence on maternal health outcomes. Several women lose their lives due to factors such as poverty, ineffective health systems and gender inequalities which make them unable to take pragmatic measures and independent critical decisions concerning their health [15].

Maternal health decisions in this study refer to those actions taken by expectant mothers, significant others, and community members in compliance with religious beliefs and practices, taboos related to pregnancy and child-birth, patronage of TBAs and herbal medications and the choice of use of orthodox health facilities and medications during pregnancy and childbirth.

Various studies have been conducted on the socio-cultural drivers of maternal mortality in Africa and elsewhere, and some interventions which are mostly determined by ‘the experts’ from outside the affected community have been put in place to address some of these factors. It is reasonable to assume that if people are aware of the socio-cultural factors and practices that increase the risk of women dying during pregnancy, childbirth and after childbirth, they will take proactive steps to curb its occurrence. This study was therefore aimed at finding out the knowledge on socio-cultural factors associated with maternal mortality (specifically TBAs, religious beliefs and practices, herbal concoctions, and pregnancy and childbirth-related taboos), and how this knowledge affects maternal health decisions, and suggest practical ways of improving upon the current maternal mortality ratio in the country from the perspectives of rural communities in the Greater Accra Region of Ghana.

## Theoretical foundation

In explaining the findings, the study is pivoted on the Health Belief Model (HBM) [15]. This model allows for comprehension and prognostication of behaviors related to health care seeking and decisions. The HBM posits that health-related behaviors are contingent on a person’s appreciation of four important and critical areas. These are: the gravity of possible illness, vulnerability to the illness, advantages of embarking on an obstructive or protective action to prevent the occurrence of the illness, and the hurdles to embarking on the action. Thus, the presupposition is that maternal health decisions are influenced by certain psychical variables and accepted modalities and norms that curtail maternal morbidity and mortality in society.

The HBM indicates that human beings are confronted with alternative actions, but would usually decide on the one perceived to be most beneficial [16]. Therefore, community members’ maternal health decisions would be the resultant effect of knowledge on socio-cultural determinants of maternal morbidity and mortality, the benefits and importance placed on some of these determinants, the perceived severity of the effects of the determinants, the witnessing of maternal morbidity and mortality in communities, belief in the abolishment of some determinants as the right course of action, and the social and economic consequences of new maternal health-related decisions.

## Methods

### Study design, area, and participants

The study was a cross-sectional one that employed mixed-method of data collection to test association between knowledge on socio-cultural factors related to maternal mortality (such as taboos related to pregnancy and childbirth, patronage of TBAs, use of herbal preparation and concoctions, and religious beliefs and practices) and maternal health decisions in three rural districts in the Greater Accra Region.

The Greater Accra Region was selected purposively for its cosmopolitan nature. The region houses people from diverse social and cultural backgrounds throughout the country, hence, access to eligible participants was relatively easier. Again, the region, according to the Ghana Health Service recorded the highest number of 201 institutional maternal deaths in the country in 2013. Purposive sampling was employed to select 3 rural districts out of the 10 districts in the region namely: La Nkwantanang-Madina, Shai Osudoku, and Accra Metropolitan for the study. Four communities were also selected from each of the selected 3 districts making a total of 12 communities. Two hundred and forty (240) participants were recruited conveniently for the study (20 each from the 12 communities), based on their age (18 years and above), number of years of stay in the community (minimum of 2 years), and willingness to participate voluntarily in the study for the quantitative study. Participants were selected from the study communities with assistance from community representatives.

Two research assistants with master of philosophy (MPhil) degrees and enormous experience in empirical research data collection assisted in the collection of data. The aim and objectives, as well as the methods of data collection for the study were thoroughly explained to them before the commencement of data collection.

For the qualitative part of the study, six (6) focus group discussions (made up of 8–10 persons per group) were organized, two for each of the 3 selected districts. The participants consisted of adult volunteers who were residents in the selected communities and were fluent in English, Ga, or Twi local languages. These participants were also assembled with assistance from community representatives. The FGDs were conducted by the author, and were used to collect spontaneous information on socio-cultural factors that influence maternal health, morbidity and mortality.

### Study instruments and data

Structured interview questionnaires were developed with the study objectives as a guide and used to collect the quantitative data. Two hundred and forty (240) self-administered questionnaires were administered to eligible participants identified with assistance from community representatives by the two research assistants and the author. Two hundred and thirty-three (233) questionnaires were fully completed and analyzed representing a response rate of 97%. The questionnaires gathered data on socio-demographic background of participants, knowledge on social and cultural norms and practices such as taboos related to pregnancy and childbirth, patronage of TBAs, use of herbal preparation and concoctions, and religious beliefs and practices as factors related to maternal morbidity and mortality and whether this knowledge influences decisions on maternal health (Additional file 1).

The qualitative data consisted of responses from focus group discussions (FGDs). FGD guide (Additional file 2) was developed to gather spontaneous or non-demand driven and general information on maternal-related morbidity and mortality, prevailing social, cultural, traditional and economic norms and practices related to maternal mortality, and how these influence decisions on maternal health and practices. The instruments for data collection were pre-tested in the Ga East district of the Greater Accra Region to determine their appropriateness for collecting the desired data. This exercise was used to test clarity, suitability as well as the logical flow of questions, and also helped in adapting the tools to the study objectives.

The FGDs were conducted in Ga or Twi local languages at convenient settings in the selected communities. The author is fluent in Ga, Twi and English, and has enormous experience in moderating FGDs in various locations in the Greater Accra Region and elsewhere.

### Data processing and analysis

The quantitative data consisted of responses from the questionnaires, and were analyzed with SPSS version 18.0. The data being categorical and nominal, the Fisher’s Exact Test of independence was first used to determine the association between knowledge on socio-cultural factors related to maternal mortality (independent variables) and maternal health decisions (dependent variable) at 95% confidence interval. Odds ratio (OR) was determined on the binary results of the ‘yes’ and ‘no’ responses to determine the magnitude of association between each of the independent variables and the dependent variable (Table 4).

For the qualitative data, audio recorded responses and notes taken from the FGDs were translated verbatim in English language and transcribed in a word processing application. The transcripts and the original recordings were reviewed several times. Key cultural terminologies were kept in the local languages. The transcripts and field notes were stored as files. Manual coding was used to select themes based on the research tools consistent with the principles of grounded theory [17]. Coding was done by placing blocks of text into nodes based on the themes. Information based on the themes were compared across the transcripts using similarities and differences in views of participants on social and cultural factors and practices affecting maternal morbidity and mortality. Quotes were then selected verbatim to instantiate the themes with minor editions to enhance readability.

## Results

### Socio-demographic characteristics of respondents

As depicted in Table 1, about 51% (118/233) of respondents for the quantitative study were females whilst about 49% (115/233) were males. The majority of respondents (93.6%; 218/233) were between the ages of 18 and 47 years, one was 70 years old. Forty-five percent (105/233) of respondents had tertiary education, 48% (112/233) were secondary school graduates (junior high or senior high) and the remaining had either primary or no formal education. The majority (about 53%; 123/233) were married, 42% (98/233) had never married before, and the rest (5%; 12/233) had no spouses because they were either widowed, separated, or divorced. Whilst only 6% (14/233) of respondents were employed in the formal sector, the overwhelming majority (82.3%; 192/233) were unemployed or artisans with the remaining 11.6% (27/233) working in the informal sector. About 88% (206/233) of respondents were Christians with only 11.6% who were either Muslim or affiliated with other religions.

**Table 1:** Socio-Demographic Background Characteristics of Respondents for quantitative study

Attribute	Frequency	Percent
Sex		
Male	115	49.4
Female	118	50.6
Age (Years)		
18–27	96	41.2
28–37	89	38.2
38–47	33	14.2
48–57	8	3.4
58–67	6	2.6
68 and above	1	0.4
Educational Background		
No Formal Education	4	1.7
Primary	12	5.1
Junior High	51	21.9
Senior High	61	26.2
Tertiary	105	45.1
Marital Status		
Married	123	52.8
Never Married	98	42.0
Divorced	6	2.6
Separated	2	0.9
Widowed	4	1.7
Employment Status		
Formally Employed	14	6.0
Informally Employed	27	11.6
Artisans	81	34.7
Unemployed	111	47.6
Religion		
Christian	206	88.4
Muslim	25	10.7
Other	2	0.9

*n* = 233

### Knowledge on socio-cultural factors associated with maternal mortality

Whilst about half (118/233; 50.7%) of respondents involved in the quantitative study were aware of TBAs as a possible socio-cultural factor related to maternal mortality in Ghana, 115 representing 49.3% did not know or were not sure about that fact. The majority (64.4%; 150/233) of respondents knew about religious beliefs and practices as associated with maternal mortality, 23.6% (55/233) of them did not know about that, and the remaining 12% (28/233) were not sure if religious beliefs and practices could cause a woman to lose her life during pregnancy and childbirth. With regard to herbal concoctions as a factor related to maternal mortality, 60.5% (141/233) of respondents knew that for a fact, 16.3% (38/233) had no knowledge on that and 23.2% (54/233) were not sure. Almost 50% (116/233) of respondents indicated knowledge on pregnancy and childbirth related taboos as a socio-cultural factor related to maternal death, whilst the other 50% (117/233) did not know or were not sure about that (Table 2).

**Table 2:** Knowledge on Socio-cultural Causes of Maternal Mortality

Knowledge on TBA patronage as possible cause of maternal mortality	Frequency	Percent
Yes	118	50.7
No	90	38.6
Not Sure	25	10.7
Knowledge on religious beliefs and practices as possible cause of maternal mortality		
Yes	150	64.4
No	55	23.6
Not Sure	28	12.0
Knowledge on herbal concoctions use as possible factor associated with maternal mortality		
Yes	141	60.5
No	38	16.3
Not Sure	54	23.2
Knowledge on pregnancy and childbirth related taboos as possible cause of maternal mortality		
Yes	116	49.8
No	78	33.5
Not Sure	39	16.7

*n* = 233. TBA – Traditional Birth Attendant

### Knowledge on socio-cultural factors associated with maternal mortality and maternal health decisions

The quantitative study as indicated in Table 3, showed statistically significant relationship between knowledge on TBA patronage, religious beliefs and practices, herbal concoctions, and pregnancy and childbirth-related taboos as factors related to maternal mortality and maternal health decisions (*p* = 0.001) for all variables. Strong associations also exist between maternal health decisions and knowledge on TBA patronage, and religious beliefs and practices as factors related to maternal mortality (OR = 7.28; OR = 21.06 respectively), whereas there is weak association between knowledge on herbal concoctions use as a factor related to maternal mortality and maternal health decision (OR = 0.78) (See Table 4).

**Table 3:** Knowledge on Socio-cultural factors related to maternal mortality and maternal health decision

Knowledge on TBA patronage as factors related to maternal mortality	Maternal health decision			*P*- value
	yes	No	Not sure	0.001
Yes	66	4	16	
No	24	19	29	
Not Sure	7	2	2	
Knowledge on religious beliefs and practices as factors related to maternal mortality				
	Yes	No	Not Sure	0.001
Yes	63	7	36	
No	21	17	8	
Not Sure	15	0	11	
Knowledge on herbal concoctions use as factors related to maternal mortality				
	Yes	No	Not Sure	0.001
Yes	68	20	13	
No	13	3	8	
Not Sure	16	3	30	
Knowledge on pregnancy and childbirth related taboos as factors related to maternal mortality				
	Yes	No	Not Sure	0.001
Yes	67	5	22	
No	14	22	21	
Not Sure	19	1	14	

*n* = 233. TBA – Traditional Birth Attendant

**Table 4:** Knowledge on Socio-cultural determinants of maternal mortality and maternal health decision

Attribute	Maternal health decision		
	OR	95% CI	*P*-value
Knowledge on TBA patronage as factors related to maternal mortality			
No	1		
Yes	13.06	3.44–49.50	0.001
Knowledge on religious beliefs and practices as factors related to maternal mortality			
No	1		
Yes	7.28	2.41–22.01	0.001
Knowledge on herbal concoctions use as factors related to maternal mortality			
No	1		
Yes	0.78	0.20–3.05	0.001
Knowledge on pregnancy and childbirth related taboos as factors related to maternal mortality			
No	1		
Yes	21.06	5.16–85.77	0.001

*TBA* – Traditional Birth Attendant

## Findings from focus group discussion

### Socio-demographic characteristics of FGD participants

Fifty-eight persons participated in the six (6) FGDs. They were made up of 21 (36.2%) males and 37 (63.8%) females, their ages ranged from 28 to 73 years. Thirty-five (60.3%) were married whilst the remaining 23(39.3%) were either single or divorced. Forty- three (74.1%) had either undergone primary or secondary education, whilst fifteen (25.9%) had no formal education.

### Factors perceived to be associated with mortality and maternal health decisions

The FGDs revealed mixed reactions among participants about the relationship between knowledge on socio-cultural factors associated with maternal mortality and maternal health decisions. Some participants believe that there is inverse relationship between knowledge on some of the socio-cultural factors related to maternal mortality and maternal health decisions whilst others were of the view that some of the factors had direct relationship with maternal health decisions.

Participants enumerated a number of socio-cultural factors as promoting maternal health or causing maternal morbidity and mortality and thus, affecting maternal health decisions under the themes: Traditional medicine, Pregnancy and Childbirth Related Taboos, Traditional Birth Attendants, Religious Beliefs and Practices, and Other Socio-Cultural Factors.

### Traditional medicine

Traditional medicine, according to the majority of FGD participants in the Accra Metropolitan area and some participants in the other study districts, is good for maternal health and safe delivery of a healthy child, and would therefore encourage pregnant women to rely on traditional medication for safe delivery. Some participants indicated that because traditional medicine is well known and accepted in the villages, maternal mortality does not occur in the villages, they only hear of it in the city where some young women refuse traditional medicine, and end up losing their lives during pregnancy or childbirth. Thus:

*“Village people don’t die during pregnancy or childbirth, we don’t even get ill, because we know all the traditional medications for various types of illnesses, and we use them when we don’t feel well, and they work very well, a woman who dies in the course of pregnancy or child birth is cursed.”*

*“No woman or child died in our family when my mother was alive, all our children were born at home, we only used traditional herbs when we’re pregnant or when we’re ill, and it worked very well for us”.*

*“My grandmother told me that when she gave birth to my mother there were no hospitals; my mother even told me that she never attended any antenatal clinic but she was very healthy and I was very strong when I was born, all the medications she depended on were traditional herbs”.*

Some participants also believe that use of traditional medication during pregnancy is dangerous to the health of the expectant mother when taken from different sources or herbalists. They indicated, however, that traditional medicine can be taken safely with orthodox medications when they are taken with some time interval apart. A participant opined that:

“*Two traditional medications from different sources don’t match, but one can use traditional and Western medicines together without any problems, problem occurs when they are taken simultaneously or instructions are not adhered to.”*

*“In Achimota, a pregnant woman died after taking hospital medication and herbal medicine at the same time, another also died because she was taking more tablets than the prescribed dose because she felt the tablets were too small in size and needed to take more for them to work.”*

### Pregnancy and childbirth-related taboos

FGD participants mentioned a number of taboos related to pregnancy and childbirth in their communities. These are related to food and where it is eaten, drinks and social norms and practices. Participants indicated that pregnant women should not eat outside the home or in public, since some evil individuals with ‘evil eye’ can kill the baby in the womb by looking at the food, and this can lead to the pregnant woman losing her life.

*“This evil person when he/she likes the pregnant woman will eat some of her food to offset the problem*.”

Some of the participants also explained that in their communities, a pregnant woman is forbidden from eating snails and crabs as she would give birth to a child with watery mouth if she ate them:

“*It is a taboo for a pregnant woman to eat snails in our community, we don’t want a child with watery mouth in our family- we are special, you know*!”

Other forbidden foods are okro, eggs, sugary and alcoholic drinks as well as smoking. These are believed to affect both the health of the unborn child and the mother.

“*A pregnant woman in my village is never allowed to eat okro, she’s also not allowed to smoke or take in sugary and alcoholic drinks, because we believe these are injurious to her and the unborn baby*”.

*“If an expectant mother eats eggs, she gives birth to a child who will become a thief when he/she grows up.”*

Some participants from northern Ghana also indicated that pregnant women, in their culture are not supposed to enter a room with their back as the child’s presentation during labor would be breach, and the woman can lose her life in the process. Again, expectant mothers are not allowed to bend and use their mouth to fan fire when cooking as:

“*Water will enter into the baby’s nose and die, and this might lead to the death of the expectant mother as well.”*

### Religious beliefs and practices

Some study participants indicated that a proportion of expectant women believe that certain illnesses suffered by expectant mothers are attributable to paranormal causes or occur as a result of some malevolent spirits, hence, such illnesses can only be cured by spiritual healers, they therefore hop from one spiritualist to another until they lose their lives.

FGD participants indicated that some religious groups do not allow their members to take medication from the hospital, and would therefore not allow the expectant mother who is ill to take any kind of medication. The Jehovah’s Witnesses were cited as not allowed to receive blood transfusion and for that matter, an anemic expectant mother would be left to die than to be transfused. It is also believed that women involved in adultery, those who disrespect their husbands, and those involved in extra-marital affairs usually lose their lives in the process of childbirth because of their sins. A participant narrated:

“*In my culture, if a married woman is impregnated by another man, she will not be able to deliver safely, and will be made to confess her sins before she can deliver the baby. If she refuses to confess, she will die*.”

Some participants also indicated that certain chiefs in their locality use pregnant women for rituals and these women die during childbirth:

“*A chief used a pregnant woman for rituals so she died during delivery, when the husband got to know about it, he also performed another ritual to curse pregnant women in the village, thus, women continued to experience pregnancy-related deaths until the gods of the land were pacified.”*

### Traditional birth attendants (TBAs)

FGD participants in the study districts explained that high poverty levels among the local people adversely influence maternal health decisions of most of the pregnant women and their families, consequently, a number of them often find it difficult to attend antenatal care during pregnancy. Thus, they depend on TBAs as alternative source of antenatal care and in some cases lose their lives, because most of these TBAs do not have the technical know-how in managing pregnancy-related complications.

“*I know a woman who spent almost two days with a TBA when she was in labor, but could not deliver at the TBA’s end, so she was transported to the hospital facility nearby, but lost her life because it was too late*.”

Others also felt that because the TBAs have experience in attending to deliveries, they are safe and their fees are reasonable. Again, most of their patrons have trust and confidence in them, since they tend to relate better to them than orthodox health care facility staff.

*“My Mother’s friend is a TBA, and she is my midwife, she’s seen me through three deliveries safely, she is a nice person and would offer advice and provide a helping hand in taking care of my babies, so if I have to deliver again, I will patronize her. Her charges are also moderate.”*

### Other socio-cultural factors

#### Laziness during pregnancy

Four of the six FGDs mentioned laziness during pregnancy as a cause of maternal mortality. Participants believed in active work during pregnancy, and explained that exercise is good and keeps mother and baby healthy and safe:

“*The main cause of maternal death is laziness, if you sleep most of the time during pregnancy, the child will not move during delivery and will make it difficult for you to deliver and even lose your life in the process, but if you exercise, you can freely deliver*.”

“*If the expectant mother is lazy, the baby in utero also becomes lazy and will not move during delivery, this makes it difficult for her to deliver safely.”*

#### Infidelity and disrespect on the part of the woman

An FGD participant who hails from the northern part of the country indicated that disrespect and infidelity on the part of the expectant mother can cause her to lose her life during childbirth. Hence, if a woman is suspected to have committed adultery, and she experiences complications related to childbirth, the only remedy to save her life and that of the unborn baby is to confess her infidelity in the presence of her husband and family members and ask him for forgiveness. According to them, a woman who is insolent towards her husband does not have it easy with childbearing.

“*In the north, an expectant woman who engages in extra-marital affair would lose her life and that of the baby during childbirth unless she kneels down on a cement block in the presence of the husband and other family members and begs for forgiveness from the husband, and once the husband accepts her apology, she can then deliver safely*.”

#### Power relations in the family

Study participants explained that power relations between mother and child and or husband and wife could determine whether a pregnant woman would attend antenatal clinic or not. Some mothers of expectant women would ensure that they provide medications for their expectant daughters, because they believe that they have experience with regard to what would make the unborn baby and the mother healthy and would even decide on the kind of foods they should take. Some husbands according to participants would also choose a healthcare provider for their expectant wives, and for those women who are economically weak, the husbands’ decisions are bound on them in all aspects of their lives.

“*I know of a very domineering husband who because the wife and her family are financially weak, takes decisions on every activity in the home. This man took some herbs from a TBA for his pregnant wife and this had severe health complications for the woman to the extent that she nearly lost her life and that of the unborn baby*.”

#### Rapport between health professionals and patients

The study revealed that where the connection between the health professional, especially the nurse and the expectant mother is smooth, it encouraged the latter to frequently attend antenatal clinics for regular check-ups. On the contrary, where the health professional fails to create a congenial atmosphere in the health facility for the expectant mother to feel at home, the expectant mother would not be impressed to go for regular reviews, and this can affect the health of the unborn baby and the mother. A participant who was an expectant mother insinuated that:

“*Some of the nurses are rude to us, and not friendly. They quite often shout at us when they feel we are not doing what they’ve asked us to do. This makes us lose interest in attending antenatal clinics, and when that happens, we feel reluctant to visit the clinic when we are in labor”*.

## Discussion

The lifetime risk of a woman in Sub-Saharan Africa dying as a result of pregnancy or childbirth is 1 in 39, compared to 1 in 4700 in developed countries [1]. Approximately 830 women die from preventable causes related to pregnancy and childbirth on a daily basis, 99% of these deaths occur in low and middle-income counties [18]. Maternal mortality, a largely avoidable cause of death, is an important focus of international development efforts, and a target for Sustainable Development Goal (SDG) 3.1. Being a global as well as a human right issue, maternal mortality is considered a violation of the rights of women and its rate is perceived as a critical index of the level of development of a country. Consequently, nations the world over have instituted programs and policies within their available resources to combat this menace. The World Health Organization (WHO) launched the Safe Motherhood Initiative (SMI) in 1987 with the aim of ensuring that women around the globe will have access to minimum basic standard obstetric care [19]. A report on the progress on the MDGs indicates that though some reduction in maternal mortality has been realized, the MDG 5 was off target in many countries, especially in low and middle-income countries [20].

Among the regions of the world, Sub-Saharan Africa records the highest with 500 maternal deaths per 100,000 live births followed by south Asia with 220 deaths per 100,000 live births [21]. The high number of maternal deaths in some areas of the world shows inequities in access to, and use of quality healthcare- an important proximate determinant of maternal mortality, and highlights the gap between the rich and the poor.

Ghana’s maternal mortality ratio currently stands at 319 per 100,000 live births [22], the situation is unacceptable especially at a time that studies have identified the causes. The problem is not only because the socio-cultural context of maternal morbidity and mortality are unknown, and therefore nothing is being done by politicians and policy makers, but partly because to a large extent, some women are economically weak, educationally disadvantaged and their voices and cries do not reach anywhere in society [4]. Various maternal health organizations have tried in their own small ways to effect change, but socio-cultural beliefs and practices surrounding maternal health and death in Ghana especially in rural settings are deeply rooted in ideologies and behaviors that are very difficult to conquer as revealed by this study.

It is evident from the quantitative study that knowledge on socio-cultural drivers of maternal mortality definitely influences the direction of maternal health decisions. For instance, knowledge on TBA patronage as a factor related to maternal mortality has a very strong association with maternal health decisions (OR = 13.06), however, reports indicate that over 30% of Ghanaian women residing in rural areas do not have access to skilled attendants during delivery [23, 24]. For these women, accessing the services of TBAs and other traditional practitioners like herbalist, diviners and spiritualist even with life-threatening obstetric complications may be their first choice for care [25–27]. It has also been found that some mothers do not follow referrals for management of obstetric complications due to fear of maltreatment from healthcare providers, lack of transportation and costs of treatment [28, 29].

The study also found strong association between knowledge on pregnancy-related taboos as a factor related to maternal mortality and maternal health decisions (OR = 21.06). However, the qualitative findings indicate clear lack of knowledge on the true and real effects of pregnancy and childbirth-related ‘dos and don’ts’ on the health outcomes of the expectant mother. Pregnancy and childbirth related taboos especially on foods as found in the qualitative study may affect the health of the expectant mother since some nutritional factors that may be critical to the health of the woman may be avoided at a time that she needs them most. Sadly, some factors of maternal mortality are ignorantly attributed to the violation of these taboos, hence, the right mode of intervention is often missed.

The role traditional herbal medicines and healers play in maternal morbidity and mortality in Ghana have been studied by some scholars, hence, a good understanding of why communities believe in them and patronize them exists. Their accessibility and availability in the community are guaranteed, communities have built trust in them, as such they are widely accepted, and when it comes to cost, they are affordable [27–29]. The weak but statistically significant association between knowledge on herbal concoctions as a factor related to maternal mortality and maternal health decisions as indicated by both the quantitative (OR = 0.78) and the qualitative study, is an attestation that the majority of Ghanaians use herbal or complementary and alternative medicine (CAM) in the treatment of various ailments [30]. This may be suggestive for the basis for exploring safe and innovative ways of integrating traditional Ghanaian medicine into orthodox medicine.

Religious beliefs and practices as a factor related to maternal mortality is found to have a strong association with maternal health decisions (OR = 7.28), yet some expectant mothers and their families especially in rural Ghana prefer the services of spiritualists and diviners for their obstetric needs and complications even when health facilities are available [31–33]. This indicates the degree of importance placed on religion by Ghanaians, and some of these beliefs and practices may only be neutralized through extensive persuasive education involving religious leaders where possible.

Ghanaian communities as portrayed in the qualitative study are saddled with so many misconceptions about the real causes of maternal illnesses and deaths that it is very difficult for the majority of the most affected communities to make the right decisions regarding maternal health, and until this situation is corrected, the menace would continue to persist with its attendant problems.

One cannot talk about the problem of maternal mortality in Ghana without the mention of functional healthcare systems. These healthcare systems are woefully inadequate in the country, not enough in the cities, let alone the rural areas. The geographical distribution of Emergency Obstetric and Newborn Care Services (EmONC) in the country is unacceptable by international standards [34]. It can be safely inferred that the absence of these structures with adequately resourced staff and equipment has contributed to the findings of the present study with regard to factors associated with maternal mortality in the country.

Efforts should be made by government and all other stakeholders in establishing well-resourced healthcare facilities in rural communities and well-structured plans for educating the public on the socio-cultural factors associated with maternal morbidity and mortality to help tackle the maternal mortality menace. Healthcare professionals especially nurses and nurse assistants should be trained in customer care and must also be sensitized on the legal issues surrounding their profession so as to enable them render their services professionally, efficiently and politely [35]. There is therefore an urgent need for political commitment on the issue of maternal mortality in Ghana. If Ghanaian political leaders will agree with Tenon (1788) that ‘No one is more worthy of care than the pregnant woman who carries within her the support of empires and the gem of future generations’, active steps would be taken to save the lives of our mothers.

## Conclusion

There are several misconceptions surrounding maternal healthcare that potentially impact on maternal morbidity and mortality in Ghana as indicated by the study. For meaningful and successful interventions on maternal mortality to be achieved, maternal health stakeholders should tackle these misconceptions on the socio-cultural factors associated with maternal mortality especially in rural areas of the country, in tandem with provision of facilities and human resource relevant to institutional care. This should be done consistently for sustained effect.

## Additional files

Additional file 1: Questionnaires- Socio-Cultural Factors Associated with Maternal Mortality. (DOCX 18 kb)

Additional file 2: FGD Guide- Socio-Cultural Factors Associated with Maternal Mortality. (DOCX 15 kb)

## Abbreviations

CAM: Complementary and Alternative Medicine
FGDs: Focus Group Discussions
HBM: Health Belief Model
MDG: Millennium Development Goal
SMI: Safe Motherhood Initiative
TBAs: Traditional Birth Attendants
UN: United Nations
UNFPA: United Nations Fund for Population Activities
UNICEF: United Nations International Children’s Emergency Fund
WHO: World Health Organization
