# Supplementary material for: Does knowledge on socio-cultural factors associated with maternal mortality affect maternal health decisions? A cross-sectional study of the Greater Accra region of Ghana
Source: BMC Pregnancy Childbirth. 2019 Jan 28;19:47. doi: 10.1186/s12884-019-2197-7 (PMC6350397; doi:10.1186/s12884-019-2197-7)
Supplement: Supplementary file 1 — Questionnaires- Socio-Cultural Factors Associated with Maternal Mortality. (DOCX 18 kb) [file 12884_2019_2197_MOESM1_ESM.docx]

**QUESTIONNAIRES ON SOCIO-CULTURAL FACTORS ASSOCIATED WITH MATERNAL MORTALITY AND MATERNAL HEALTH DECISIONS**

**REGION: GREATER ACCRA**

| Date |  |
| --- | --- |
| Interviewer’s Name |  |
| Interviewer’s Remarks |  |
| Interviewer’s Signature |  |
| Time |  |

Name of District/Metropolis: ……………………………………………

Name of community: …………………………………………….

**Section A: Socio-Demographic Background Information on Interviewees**

1. Gender: **1.** Male **2**. Female
2. Age:  **1**. 18-27 **2**. 28-37 **3**. 38-47 **4**. 48-57 **5**. 58-67 **6**. 68+
3. How long have you stayed in this community? **1.** 2-6 years **2**. 7-11 years **3**. 12 years +
4. Formal educational level attained: **1**. None **2**. JHS **3**. SHS **4**. Tertiary **5**. Post graduate
5. Religious affiliation: **1**. Christianity **2**. Islam **3**. Traditional **4**. Other
6. Marital Status: **1**. Married **2**. Never Married **3**.Divorced **4**. Separated **5**. Widowed
7. Ethnicity: **1**. Ga **2**. Akan **3.** Other ………………………….. (specify)
8. Employment Status? **1**. Formally Employed **2**. Informally Employed **3**. Artisan **4**. Unemployed

**Section B: Knowledge on Socio-Cultural Determinants of Maternal Morbidity and Mortality and Maternal Health Decisions**

1. Which of the following socio-cultural practices affect maternal health? **1**. Taboos and Spiritual Practices **2.** Traditional Midwife **3**. Smoking/Drinking Alcohol/Bad Health Behavior **4**. Other …………………………………….. (specify)
2. Is TBA patronage a possible cause of maternal death? **1**. Yes **2.** No **3**. Not Sure
3. Will your answer to question **10** influence your choice of health facility for an expectant mother? 1. Yes 2. No 3. Not Sure
4. Can religious beliefs and practices cause maternal death? **1**. Yes **2**. No 3. Not Sure
5. Will your answer to question **12** influence your choice of health facility for an expectant mother? **1.** Yes **2**. No **3**. Not Sure
6. Can the use of herbal preparations by expectant mothers cause maternal death? **1**. Yes **2**. No **3**. Not Sure
7. Will your answer to question **14** influence your choice of health facility for an expectant mother? **1**. Yes **2**. No **3**. Not Sure
8. Can pregnancy and childbirth-related taboos cause maternal death? **1**. Yes  **2**. No **3**. Not Sure
9. Will your answer to question **16** influence your choice of health facility for an expectant mother? **1**. Yes **2**. No  **3**. Not Sure

***THANKS SO MUCH FOR YOUR TIME***
