# Supplementary material for: Does knowledge on socio-cultural factors associated with maternal mortality affect maternal health decisions? A cross-sectional study of the Greater Accra region of Ghana
Source: BMC Pregnancy Childbirth. 2019 Jan 28;19:47. doi: 10.1186/s12884-019-2197-7 (PMC6350397; doi:10.1186/s12884-019-2197-7)
Supplement: Supplementary file 2 — FGD Guide- Socio-Cultural Factors Associated with Maternal Mortality. (DOCX 15 kb) [file 12884_2019_2197_MOESM2_ESM.docx]

**FOCUS GROUP DISCUSSION (FGD) GUIDE ON SOCIO-CULTURAL FACTORS ASSOCIATED WITH MATERNAL MORTALITY AND MATERNAL HEALTH DECISIONS**

**Demographic Information on Participants**

1. Number of participants ___________
2. Number of males _______________
3. Number of Females ___________
4. Age range of participants ___________
5. Number of married participants ___________
6. Number of unmarried participants __________
7. Number of divorced participants _____________
8. Number of participants with no formal education _________
9. Number of participants with primary education __________
10. Number of participants with secondary education ___________
11. Number of participants with tertiary education __________

**Information on Social and Cultural Practices Associated with Maternal Mortality**

1. Prevailing social activities, economic, traditional and cultural practices in community
2. Social, economic, traditional and cultural practices associated with maternal health and mortality
3. How Social, economic, traditional and cultural practices affect maternal mortality and maternal health decisions
4. Effects of maternal illness and death on families and communities
5. Suggestions on how to prevent maternal illness and deaths with respect to social activities and cultural practices.
